# MBNL1-mediated alternative splicing in cancer: underlying mechanism, isoform regulation, and translational perspectives

**DOI:** 10.3389/fmolb.2026.1867214

**Published:** 2026-06-11

**Authors:** Huidan Tan, Rongyan Zhao, Bo Liu, Leilei Fu

**Affiliations:** 1 Department of Pharmacy, Cancer Center and State Key Laboratory of Biotherapy, West China Hospital, Sichuan University, Chengdu, China; 2 Sichuan Engineering Research Center for Biomimetic Synthesis of Natural Drugs, School of Life Science and Engineering, Southwest Jiaotong University, Chengdu, China

**Keywords:** alternative splicing, cancer, isoform regulation, MBNL1, RNA-binding protein

## Abstract

Alternative splicing (AS) is a major mechanism that expands proteomic diversity and fine-tunes gene expression in eukaryotic cells. Its dysregulation is now recognized as a hallmark of cancer and contributes to tumor initiation, progression, metastasis, and therapeutic resistance. Muscleblind-like splicing regulator 1 (MBNL1) is a highly conserved RNA-binding protein (RBP) that controls AS, RNA stability, and other aspects of transcript processing. Increasing evidence indicates that MBNL1 expression, isoform composition, and subcellular localization are frequently altered in multiple cancer types. Through these changes, MBNL1 reshapes the splicing programs of cancer-related genes and exerts context-dependent tumor-suppressive or tumor-supportive effects. This review summarizes the structure and biological functions of MBNL1, the major mechanisms through which it regulates cancer-associated AS, its expression and isoform-specific features across different tumor types, and emerging therapeutic strategies targeting MBNL1 and its downstream splicing network. Current challenges and future directions are also discussed. Overall, MBNL1 represents a promising splicing regulator with potential value for biomarker development and precision cancer therapy.

## 1 Introduction

Alternative splicing (AS) is a major post-transcriptional regulatory mechanism that enables a single gene to generate multiple transcript and protein isoforms, thereby markedly expanding transcriptomic and proteomic complexity (Tao et al., 2024). In humans, most multi-exon genes undergo AS, which is governed by a highly coordinated network of cis-regulatory elements and trans-acting splicing factors, with RNA-binding proteins (RBPs) playing essential roles in splice-site selection and spliceosome assembly (Wang and Aifantis, 2020; Choi and Thomas-Tikhonenko, 2021). Mutations in splice sites, alterations in RNA structure, spliceosome dysfunction, and aberrant expression or activity of RBPs can all disrupt AS homeostasis, leading to the production of abnormal transcripts and protein isoforms and contributing to the pathogenesis of multiple diseases, particularly cancer (Zhang et al., 2021; Bradley and Anczuków, 2023).

Aberrant AS may provide selective advantages to tumor cells because it enables rapid transcriptomic and proteomic diversification without requiring permanent genomic alterations. By altering exon inclusion, splice-site usage, or untranslated regions, tumor cells can generate isoforms that enhance survival, stress adaptation, invasion, immune escape, and drug resistance. In this sense, AS dysregulation acts as a molecular plasticity mechanism that allows cancer cells to adapt to hypoxia, metabolic stress, therapeutic pressure, and changing tumor microenvironmental conditions (Wang and Aifantis, 2020; Zhang et al., 2021; Bradley and Anczuków, 2023).

Among the RBPs involved in this process, Muscleblind-like splicing regulator 1 (MBNL1) has attracted increasing attention (Konieczny et al., 2014; Biswas et al., 2024; Zhou et al., 2025). MBNL1 was first studied in myotonic dystrophy (DM), where its sequestration by toxic CUG-repeat RNA causes widespread splicing defects (Soltanzadeh, 2022). More recently, MBNL1 has been implicated in many solid and hematologic malignancies (Sebestyén et al., 2016; Zhang et al., 2022). Notably, MBNL1 does not act uniformly across cancers. Depending on tumor type, molecular subtype, cellular context, and isoform composition, it may exert either tumor-suppressive or tumor-supportive functions (Zhou et al., 2025). This functional plasticity, together with its extensive control over cancer-related transcript processing, makes MBNL1 an attractive candidate for splicing-based precision therapy. Accordingly, this mini review examines how MBNL1 dysregulation reshapes oncogenic RNA processing, how isoform-selective regulation modifies these effects, and what translational evidence currently exists. We propose a unified framework in which MBNL1 integrates upstream signaling, isoform-selective regulation, and lineage-specific RNA-processing programs, which are revisited through mechanistic, cancer-context-specific, and translational perspectives.

## 2 Structure and function of MBNL1

### 2.1 Molecular characteristics and regulatory mechanisms of MBNL1

MBNL1 is an evolutionarily conserved RBP with homologs in mammals, flies, and nematodes, highlighting its fundamental biological importance. It belongs to the MBNL family, together with MBNL2 and MBNL3, which share partial functional redundancy but differ in tissue distribution and target specificity (Pascual et al., 2006; Arandel et al., 2022).

MBNL1 recognizes target RNAs mainly through tandem CCCH zinc-finger domains that bind YGCY sequence motifs (Park et al., 2017; Taylor et al., 2018). Its binding affinity is further influenced by RNA secondary structure and surrounding sequence context (Taylor et al., 2018). Through this sequence-selective binding, MBNL1 regulates exon inclusion or skipping and thereby shapes transcript isoform output. In general, binding of MBNL1 upstream of an alternative exon tends to promote exon exclusion, whereas binding downstream often favors exon inclusion (Wang et al., 2012; Konieczny et al., 2014).

MBNL1 activity is also influenced by its own AS. Alternative exons 3, 5/6, and seven encode or modify regions with distinct regulatory functions. Although the canonical RNA-recognition activity of MBNL1 is mainly mediated by tandem CCCH zinc-finger domains that bind YGCY-containing motifs, exon 3 has been reported to enhance MBNL1 affinity for pre-mRNA target sites and splicing regulatory activity. Exons 5/6 contribute to nuclear localization, thereby affecting access to nuclear pre-mRNA substrates, whereas exon seven is associated with self-dimerization and may influence isoform-specific regulatory output. In addition, RNA structural context and motif arrangement further modulate MBNL-dependent AS activity, indicating that MBNL1 isoform function is determined by both protein structure and RNA substrate architecture (Tran et al., 2011; Botta et al., 2013; Taylor et al., 2018). Accordingly, MBNL1 should not be viewed as a single functional entity but as a set of isoforms with different regulatory capacities. Importantly, currently available cancer studies do not support treating total MBNL1 abundance as a surrogate for functional output, because exon-defined isoforms may differ in localization, target selection, and phenotypic consequences. This issue is best illustrated in colorectal cancer (CRC) and prostate cancer (PCa), where exon 5/7-associated isoforms are linked to differential apoptosis, migration, or survival phenotypes (Tabaglio et al., 2018; Chen et al., 2020). Importantly, MBNL1 can autoregulate its own splicing, forming feedback loops that help maintain splicing homeostasis (Konieczny et al., 2017). Another important feature is its interplay with CUG-BP and ETR-3 like factor (CELF) family proteins. During development, MBNL and CELF proteins act as opposing regulators of embryonic-to-adult splicing transitions, and their relative activities determine large sets of developmentally regulated AS events (Kalsotra et al., 2008; Konieczny et al., 2014; Wang et al., 2015; Dastidar et al., 2022). In cancer, reduced MBNL1 activity may weaken adult-like splicing control and favor reactivation of embryonic or fetal-like splice isoforms, thereby supporting lineage plasticity and oncogenic dedifferentiation (Sebestyén et al., 2016; Ray et al., 2020).

### 2.2 MBNL1 core functions

The best-characterized function of MBNL1 is regulation of AS, but its role extends beyond exon selection. MBNL1 also participates in RNA stability, transcript localization, 3′-untranslated region (UTR)-dependent regulation, and translation (Wang et al., 2012; Batra et al., 2014; Choi et al., 2015). Through these processes, it contributes to cell differentiation, tissue homeostasis, and disease pathogenesis (Zhou et al., 2025). These noncanonical RNA-processing functions broaden its impact on tumor biology and help explain why MBNL1-mediated regulation is often highly context-dependent.

Under physiological conditions, MBNL1 is a key regulator of the developmental shift from embryonic to adult splicing programs. It controls splicing in multiple tissues, including muscle, heart, and brain, and is required for normal differentiation. Low MBNL1 expression is characteristic of embryonic stem cells and induced pluripotent stem cells, whereas increased MBNL1 activity promotes differentiated splicing states (Kalsotra et al., 2008; Han et al., 2013; Venables et al., 2013).

Under pathological conditions, MBNL1 dysfunction is most classically linked to trinucleotide amplification diseases (Miller et al., 2000; Fernandez-Costa et al., 2011; Moreno et al., 2022). In recent years, cancer has emerged as another major disease context involving MBNL1 dysregulation. Large-scale analyses of RBPs in cancer have shown that MBNL1-associated splicing changes overlap with undifferentiated cell-like programs and affect multiple cancer drivers (Zhang et al., 2022).

## 3 Core molecular mechanisms of MBNL1-mediated AS in cancer initiation and progression

Building on the unified framework proposed in the Introduction, this section focuses on the mechanistic layer of MBNL1-mediated AS in cancer.

### 3.1 Core regulatory pathway: MBNL1 aberration mediates target gene splicing disorder

**FIGURE 1:**
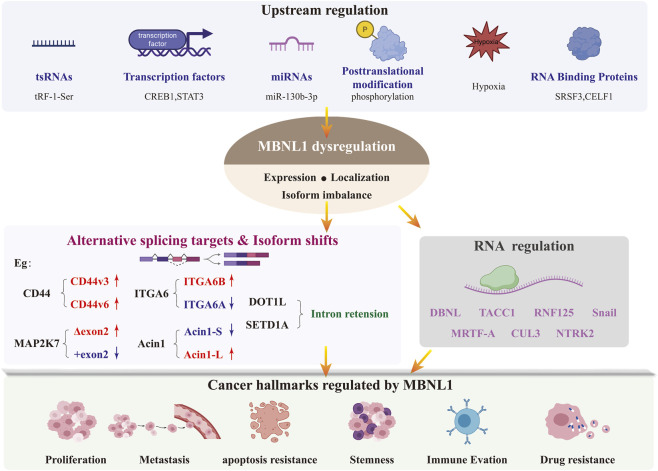
Unified conceptual model of context-dependent MBNL1 biology in cancer. Upstream regulatory signals alter MBNL1 expression, isoform composition, and subcellular localization, thereby reshaping alternative splicing and RNA-processing programs in cancer cells. These changes influence multiple malignant phenotypes, including stemness, metastasis, apoptosis resistance, immune evasion, and therapeutic response.

A central mechanism linking MBNL1 to cancer is disruption of its expression, localization, or isoform balance, which then alters the splicing of downstream targets. This can be summarized as a pathogenic cascade: MBNL1 dysregulation → target transcript mis-splicing → signaling imbalance → malignant phenotypes (Figure 1). These signaling alterations may arise from both mis-splicing of pathway-related transcripts and upstream oncogenic or stress signals that reshape MBNL1 activity. Current evidence supports context-dependent links between MBNL1-mediated RNA regulation, mTORC1-related signaling, hypoxia-associated stemness, and metabolic reprogramming (Voss et al., 2020; Li et al., 2022; Zhao et al., 2022).

When MBNL1 is reduced or functionally impaired, fetal or oncogenic splice variants may accumulate. For example, in CRC, decreased MBNL1 is associated with increased CD44 variant isoforms, particularly CD44v3 and CD44v6, which support proliferation and invasion (Navvabi et al., 2021). In leukemia, MBNL1 regulates splicing of epigenetic regulators such as disruptor of telomeric silencing 1-like (DOT1L) and SET domain containing 1A (SETD1A), thereby sustaining leukemic transcriptional programs (Itskovich et al., 2020). In PCa, altered exon seven inclusion changes the relative abundance of functionally distinct MBNL1 isoforms and influences tumor cell survival (Tabaglio et al., 2018). MBNL1 function can also be shaped by post-translational modifications and nucleocytoplasmic transport. These mechanisms affect RNA binding and target selection, further increasing the context specificity of MBNL1-mediated AS regulation (Wang et al., 2018; Liu et al., 2022; Wan et al., 2024).

### 3.2 Regulation of downstream biological effects: covering key phenotypes of tumor cells

Through its control of AS and RNA metabolism, MBNL1 influences several hallmark cancer phenotypes. In the context of proliferation and apoptosis, the key issue is not whether MBNL1 is uniformly pro- or anti-survival, but which survival-related RNA targets are engaged in a given tumor setting. Thus, altered MBNL1 activity may either weaken pro-apoptotic programs or support tumor-specific RNA-processing dependencies, producing opposite effects on tumor cell survival (Tabaglio et al., 2018; Chen et al., 2020; Itskovich et al., 2020). In CRC, altered MBNL1 activity affects the balance of apoptotic chromatin condensation inducer 1 (ACIN1) isoforms and modulates stress-induced apoptosis (Chen et al., 2020). Another study in CRC showed that, MBNL1 blocks Dicer processing of pre-miR-1307, reducing miR-1307 levels and upregulating anti-apoptotic Bcl2 (Tang et al., 2015). In glioma, impaired nuclear MBNL1 under hypoxia contributes to stem-like survival states (Voss et al., 2020).

Second, MBNL1 affects invasion and metastasis. In several solid tumors, reduced MBNL1 favors pro-invasive isoforms linked to epithelial-mesenchymal transition (EMT). MBNL1 can suppress expression of metastasis-associated transcript variants either through direct splicing regulation or by stabilizing anti-metastatic mRNAs. Loss of this restraint promotes migratory and invasive phenotypes (Tang et al., 2019; Navvabi et al., 2021; Liu et al., 2022).

Third, MBNL1 is closely connected to cancer stemness and dedifferentiation. Because it normally supports adult splicing programs, reduced MBNL1 may drive a shift toward embryonic-like isoforms associated with stem-cell traits, tumor initiation, and therapy resistance (Sebestyén et al., 2016). In glioblastoma (GBM) and other tumors, restoration of active MBNL1 suppresses self-renewal and tumor-initiating capacity (Voss et al., 2020; Zhang et al., 2022; Rendina et al., 2026).

### 3.3 Upstream regulation and synergistic effects: improving the regulatory network

MBNL1 itself is regulated by multiple upstream signals, including microRNAs, transcription factors, RNA modifications, stress-related pathways and so on. These regulators can alter MBNL1 abundance, isoform usage, localization, or binding capacity. For example, in breast cancer, tRF-1-Ser (transfer RNA-derived small RNAs (tsRNAs)) can be negatively regulated by 25-hydroxyvitamin D (25(OH)D), inhibiting MBNL1 function by blocking its nuclear transport and promoting tumor cell proliferation and stemness (Wan et al., 2024). In gastric cancer (GC), through the integration of differential network information and molecular-cell experiments, MBNL1 is differentially regulated by CREB1 during GC tumorigenesis (Yu et al., 2022). Furthermore, the signal transducer and activator of transcription 3 (STAT3)/miR-130b-3p/MBNL1 feedback loop plays a crucial role in mTORC1- mediated angiogenesis and tumor progression. MBNL1 was identified as a direct target of miR-130b-3p, and it feedback-inhibits mTORC1-activated STAT3 activation in cells (Li et al., 2022).

In other settings, MBNL1 also cooperates or competes with other splicing factors, including SRSF proteins and components of the U2 complex, which can influence both its own splicing and that of downstream targets (Tabaglio et al., 2018). In addition, emerging evidence suggests that RNA modifications and phosphorylation events may alter MBNL1 function (Wang et al., 2018; Liu et al., 2022). For example, methyltransferase 3, N6-adenosine-methyltransferase complex catalytic subunit (METTL3) -mediated m6A modification regulates cyclin-dependent kinase 12 (CDK12) activity through BUD13, thereby promoting phosphorylation of MBNL1 at the Ser280 site; this modification enhances the binding of MBNL1 to fibroblast growth factor receptor 2 (FGFR2) precursor mRNA, promotes the expression of mesenchymal isoforms, drives GBM angiogenesis, and thus regulates GBM proliferation, migration, and invasion (Liu et al., 2022). Together, these upstream and cooperative regulatory layers indicate that MBNL1 activity is shaped by signaling inputs, RNA modification, noncoding RNA regulation, RBP interactions, and isoform-specific functional states.

## 4 Context-dependent splicing regulation of MBNL1 in cancer

After defining the mechanistic components of the unified framework, we next apply this framework to different cancer contexts. Across cancers, the most informative distinctions concern whether MBNL1 is functionally attenuated or maintained as a dependency, whether isoform balance is altered, and which downstream splice events best report biological activity. This pattern-based view also helps separate conceptual mechanisms (Figure 1) from representative study-level evidence (Supplementary Table S1).

### 4.1 Pattern I: loss of MBNL1 activity in solid tumors drives fetal-like and pro-invasive splicing

Across many solid tumors, the dominant theme is not simple mutation of MBNL1 but functional attenuation through reduced expression, impaired nuclear localization, or antagonism by upstream regulators. This attenuation shifts cells away from differentiated adult splicing programs and toward fetal-like or oncogenic transcript states. Representative examples include increased CD44 variant usage and ACIN1 isoform switching in CRC, MAP2K7 exon two skipping in stemness-associated programs, destabilization of invasion-suppressive transcripts such as DBNL and TACC1, and hypoxia-induced loss of nuclear MBNL1 activity in GBM (Fish et al., 2016; Chen et al., 2020; Ray et al., 2020; Voss et al., 2020; Navvabi et al., 2021).

Although the downstream targets differ by lineage, the shared biological consequences are highly convergent: enhanced invasion or EMT-like behavior, reduced apoptosis, dedifferentiation/stemness, and resistance-associated phenotypes. Thus, the common principle in solid tumors is that MBNL1 loss-of-function rewires RNA processing toward a more plastic and aggressive cellular state rather than acting through one universal target transcript (Tang et al., 2019; Liu et al., 2022; Zhang et al., 2022).

### 4.2 Pattern II: isoform-selective regulation modifies biological output

A second pattern is that total MBNL1 expression alone can be misleading, because exon-defined isoforms differ in localization and regulatory output. Exons 5/6 and seven are particularly relevant in cancer-related settings. In CRC, the MBNL18 isoform containing exons five and seven is enriched in tumor tissue and participates in the SRSF3-MBNL1-ACIN1 axis that favors survival under stress, whereas in PCa altered exon seven inclusion changes the balance between functionally distinct isoforms and influences viability and migration (Tabaglio et al., 2018; Chen et al., 2020). These studies indicate that isoform composition is not a secondary detail but a mechanistically meaningful layer that can modify the phenotypic consequence of MBNL1 dysregulation.

### 4.3 Pattern III: MBNL1 dependency in hematologic malignancies

The above solid-tumor pattern contrasts with MLL-rearranged leukemia, where MBNL1 is maintained at high levels and contributes to leukemic cell fitness. In this setting, the relevance of MBNL1 appears to arise not simply from its abundance, but from its role in maintaining leukemia-associated RNA-processing programs. For example, MBNL1 regulates alternative splicing of leukemogenic regulators such as DOT1L and SETD1A, and genetic or pharmacologic inhibition of MBNL1 impairs leukemia cell survival (Itskovich et al., 2020). This example suggests that splicing-directed therapeutic vulnerability may depend on whether a tumor lineage relies on specific MBNL1-regulated RNA-processing events, rather than on total MBNL1 expression alone.

### 4.4 Translational implications for biomarker development

Taken together, current data suggest that the most informative biomarkers will likely be composite rather than single-parameter measures. Total MBNL1 abundance, exon 5/7 usage, nuclear-cytoplasmic distribution, and selected downstream splicing events may each capture different layers of biological activity. Therefore, patient stratification should be based on functional MBNL1 states - for example, MBNL1-low solid tumors with fetal-like splicing reactivation versus MBNL1-dependent hematologic malignancies - rather than on expression level alone (Tabaglio et al., 2018; Itskovich et al., 2020; Voss et al., 2020). This functional-state-based stratification is consistent with broader precision-classification approaches that integrate transcriptomic and clinical features to support biomarker-guided therapeutic decisions (Tang et al., 2024).

## 5 Translational implications of targeting MBNL1-associated splicing networks

Based on the key regulatory role and functional specificity of MBNL1 in AS in cancer, targeting MBNL1 and its regulated splicing network has become a new direction for cancer treatment. The unified framework also has direct translational implications. If MBNL1 functions through context-dependent regulatory states rather than as a uniform oncogene or tumor suppressor, therapeutic strategies should be matched to specific MBNL1 functional states.

### 5.1 Small molecule inhibitors targeting MBNL1

Small molecules that inhibit MBNL1 are most relevant in cancers where MBNL1 acts as a tumor-supportive factor, particularly MLL-rearranged leukemia. By disrupting MBNL1-RNA interactions or its downstream splicing activity, these agents may suppress leukemic proliferation while sparing normal cells (Itskovich et al., 2020). However, current MBNL1-directed compounds remain at the proof-of-concept stage, and their translation is limited by selectivity, delivery, and the risk of perturbing physiological RNA-processing programs in normal cells. Therefore, future studies should evaluate transcriptome-wide off-target splicing effects and define biomarkers for tumors that are truly dependent on MBNL1-regulated splicing (Itskovich et al., 2020; Takayama et al., 2023; Lv et al., 2025).

### 5.2 Splice-switching oligonucleotides (SSOs) strategy

SSOs offer a more precise approach by directly modulating exon selection (Khurshid et al., 2024). In the MBNL1 field, SSOs can be designed either to correct MBNL1’s own isoform imbalance or to reverse aberrant splicing of downstream cancer-related transcripts. This strategy is particularly attractive in tumors such as PCa, where specific isoforms appear to have distinct functional consequences (Tabaglio et al., 2018). The broader success of antisense oligonucleotide-based therapies in noncancer diseases supports the translational potential of this platform, although tumor-specific delivery remains a major obstacle (Lv et al., 2025).

### 5.3 Gene therapy regulates MBNL1 expression

Gene therapy represents a direct strategy to modulate MBNL1 expression in cancer using viral or non-viral delivery systems. In GBM, restoration of active nuclear MBNL1 suppresses glioma stem cell self-renewal, inhibits tumor growth (Voss et al., 2020). In lung adenocarcinoma (LUAD), MBNL1 overexpression may enhance chemosensitivity and antitumor immunity, at least partly through stabilization of RNF125 and subsequent repression of PD-L1-related signaling (Yan et al., 2025). Conversely, in tumors with oncogenic MBNL1 dependence, such as MLL-rearranged leukemia, RNA interference-mediated MBNL1 silencing disrupts the DOT1L/SETD1A-associated splicing program, thereby inhibiting leukemic cell proliferation and promoting apoptosis (Itskovich et al., 2020). Clustered regularly interspaced short palindromic repeats (CRISPR)/CRISPR-associated protein 9 (Cas9)-based editing further supports the functional importance of MBNL1, as MBNL1 depletion in GBM models increases HIF-1α protein levels despite minimal change at the mRNA level (Rendina et al., 2026). Nevertheless, these findings remain largely proof-of-concept, and major barriers to clinical translation—including vector safety, immunogenicity, and tumor-specific delivery efficiency—still need to be overcome.

### 5.4 Combination therapy strategy

Because MBNL1 can function either as a tumor suppressor or as a cancer dependency, combination therapy is likely to be more realistic than a uniform strategy of global inhibition or activation. A rational approach is to stratify patients according to MBNL1 expression status, isoform profile, or downstream splicing signatures, and then combine MBNL1-directed interventions with pathway-targeted or standard anticancer therapies.

For example, in MBNL1-low tumors, splicing-driven dedifferentiation programs such as MAP2K7 exon two skipping may predict susceptibility to JNK inhibition (Ray et al., 2020). In LUAD, the MBNL1-RNF125 axis suggests possible synergy with immune checkpoint blockade (Yan et al., 2025). Together, these observations provide a rationale for exploring selected combinations with pathway-targeted, metabolic, epigenetic, or RNA-targeting approaches, but direct evidence for MBNL1-based combination therapy remains limited (Itskovich et al., 2020; Zhao et al., 2022; Khurshid et al., 2024). Such strategies should therefore be considered only in tumor contexts in which MBNL1-associated splicing states are mechanistically linked to the relevant pathway.

### 5.5 Emerging technologies for resolving MBNL1 splicing heterogeneity

Recent advances in single-cell and spatial transcriptomics are providing new opportunities to investigate MBNL1-mediated RNA processing with higher cellular resolution. Traditional bulk RNA sequencing may obscure cell-type-specific splicing programs and isoform diversity within heterogeneous tumors (Benegas et al., 2022; Bradley and Anczuków, 2023). This limitation is particularly relevant for MBNL1 because its activity is strongly influenced by cellular state and microenvironmental context (Ray and Epstein, 2020).

Single-cell RNA sequencing (scRNA-seq), especially long-read and isoform-resolved approaches, can identify lineage-specific splicing events and rare transcript isoforms associated with stemness and therapy resistance (Wu and Schmitz, 2023). These approaches may help clarify why MBNL1 exhibits divergent biological roles across different tumor ecosystems. In addition, computational frameworks for single-cell splicing analysis may help distinguish whether MBNL1 dysregulation acts as a driver of dedifferentiation or reflects adaptive cellular states (Ray et al., 2020).

Spatial transcriptomics further extends this framework by preserving tissue architecture and microenvironmental context. In GBM and other hypoxic tumors, spatial analyses may reveal regional differences in MBNL1 activity and stemness-associated niches (Ren et al., 2023; Haley et al., 2024). Integration of single-cell and spatial transcriptomics may therefore improve biomarker discovery and support precision targeting of tumor-specific splicing networks (Liu et al., 2026).

## 6 Discussion

Collectively, current evidence supports a model in which MBNL1 functions not as a universal oncogene or tumor suppressor, but as a dynamic regulator of cancer-associated RNA processing integrating upstream signaling, isoform-selective regulation, and lineage-specific transcriptomic programs. By revisiting this framework across mechanistic, cancer-context-specific, and translational sections, this review highlights that the biological output of MBNL1 depends on the interaction between regulatory inputs, MBNL1 functional states, and tumor-lineage-specific target repertoires.

As a central regulator of alternative splicing and broader RNA-processing events, MBNL1 influences multiple malignant phenotypes, including proliferation, apoptosis resistance, invasion, stemness, immune modulation, and therapeutic response (Wang and Aifantis, 2020; Hildebrandt et al., 2023; Gabel et al., 2025). However, its biological consequences vary substantially across tumor contexts. In many solid tumors, reduced or functionally impaired MBNL1 is associated with fetal-like splicing reactivation, dedifferentiation, and malignant progression, whereas in specific hematologic malignancies such as MLL-rearranged leukemia, MBNL1 may instead function as an oncogenic dependency (Obeng et al., 2019; Ray and Epstein, 2020; Stanley and Abdel-Wahab, 2022).

Several factors may contribute to these apparently contradictory observations. First, total MBNL1 abundance does not necessarily reflect functional activity. Nuclear-cytoplasmic redistribution, post-translational modifications, and upstream signaling pathways may alter RNA-binding behavior and target selection without substantially changing expression levels (Voss et al., 2020; Liu et al., 2022; Wan et al., 2024). Second, increasing evidence indicates that distinct MBNL1 isoforms may exert different or even opposing biological effects. Isoforms defined by exon five or exon seven inclusion differ in localization, dimerization potential, and regulatory output, thereby generating divergent phenotypic consequences across tumor contexts (Botta et al., 2013; Tabaglio et al., 2018; Hildebrandt et al., 2023). Third, the downstream consequences of MBNL1 dysregulation are highly dependent on lineage-specific transcriptomic landscapes. Different cancers express distinct repertoires of splicing-sensitive targets, producing heterogeneous biological outputs even under similar MBNL1 perturbations (Sebestyén et al., 2016; Itskovich et al., 2020).

Another unresolved issue is whether MBNL1 dysregulation primarily functions as a driver event or instead represents a secondary consequence of tumor evolution and dedifferentiation. In some tumors, fetal-like splicing programs emerge together with reduced MBNL1 activity (Sebestyén et al., 2016; Ray et al., 2020), whereas in other contexts MBNL1 alterations may occur downstream of hypoxia, m6A remodeling, or kinase-mediated signaling changes (Liu et al., 2022; Wan et al., 2024). These observations suggest that MBNL1 may function both as an active regulator of oncogenic RNA processing and as a responsive node within broader stress-adaptive signaling networks.

Current understanding of MBNL1-associated splicing regulation is still largely based on bulk transcriptomic analyses, which may underestimate intratumoral heterogeneity and isoform diversity (Benegas et al., 2022; Bradley and Anczuków, 2023). Emerging technologies, including single-cell transcriptomics, long-read sequencing, and spatial transcriptomics, are expected to provide improved resolution of lineage-specific splicing programs and context-dependent RNA-processing states (Wu and Schmitz, 2023; Liu et al., 2026). Integration of these approaches may help clarify the divergent roles of MBNL1 across different tumor ecosystems and support the development of more precise splicing-based therapeutic strategies (Li et al., 2024; Liu et al., 2026).

In summary, MBNL1 is emerging as an important regulator of cancer-associated alternative splicing with considerable translational promise. Future progress in the field will likely depend on shifting from static expression-based models toward dynamic and systems-level interpretations of MBNL1 biology. Clarifying isoform-specific functions, mapping context-dependent splicing networks, establishing robust biomarkers, and improving delivery strategies for RNA- and gene-based therapies may ultimately support the development of splicing-guided precision oncology approaches targeting MBNL1-associated RNA-processing networks.
