# Supplementary material for: MBNL1-mediated alternative splicing in cancer: underlying mechanism, isoform regulation, and translational perspectives
Source: Front Mol Biosci. 2026 Jun 11;13:1867214. doi: 10.3389/fmolb.2026.1867214 (PMC13294462; doi:10.3389/fmolb.2026.1867214)
Supplement: Supplementary file 1 [file Table1.docx]

Supplementary Table S1. Representative regulatory networks, functional consequences, and evidence basis of MBNL1-mediated RNA processing in cancer.

| **Regulatory category** | **Target/ regulator** | **Molecular mechanism** | **Functional consequence** | **Cancer context** | **Evidence/ model** | **Key reference** |
| --- | --- | --- | --- | --- | --- | --- |
| MBNL1 isoform regulation | MBNL1 exon 7 | Increased MBNL1 +exon7 isoform | Anti-apoptotic effect | Multiple cancers | Cancer cell functional assays | (Tabaglio et al., 2018) |
| AS | MAP2K7 | Increased MAP2K7 Δexon2 isoform → JNK pathway activation | Stemness maintenance and tumor growth | Multiple solid tumors | Transcriptomic analysis and functional validation | (Ray et al., 2020) |
| AS | CD44 | Increased CD44v3/ CD44v6 isoforms | Altered proliferation and invasion-related phenotypes | CRC | Patient samples/ expression-splicing association | (Navvabi et al., 2021) |
| AS | DOT1L/ SETD1A | Intron exclusion-dependent splicing regulation | Leukemia cell survival | MLL-rearranged leukemia | In vitro and in vivo leukemia models | (Itskovich et al., 2020) |
| AS | ACIN1 | Decreased Acin1-S/ increased Acin1-L isoforms | Reduced apoptosis | CRC | CRC cell functional assays | (Chen et al., 2020) |
| AS | ITGA6 | Decreased ITGA6A/ increased ITGA6B isoforms | Enhanced migration and metastasis | triple-negative breast cancer (TNBC) | Breast cancer functional models | (Seachrist et al., 2020) |
| RNA stability | DBNL/ TACC1 | 3′UTR binding-mediated transcript stabilization | Suppression of metastasis | Breast cancer | In vitro and metastasis models | (Fish et al., 2016) |
| RNA stability | Snail | Recruitment to P-bodies → mRNA decay | Inhibition of EMT | CRC | CRC cell functional assays | (Tang et al., 2019) |
| RNA stability | RNF125 | MBNL1-mediated mRNA stabilization | Linked to immune response modulation | LUAD | LUAD cell assays and immune-related analysis | (Yan et al., 2025) |
| RNA stability | CUL3 | 3′UTR binding-mediated transcript stabilization | Increased drug sensitivity | Cervical cancer | Cervical cancer cell model | (Wang et al., 2020) |
| RNA stability | MRTF-A | mRNA stabilization | Associated with altered migration | Esophageal cancer | Esophageal cancer cell model | (Liang et al., 2022) |
| circRNA regulation | circNTRK2 | Regulation of circRNA biogenesis | Inhibition of glycolytic reprogramming | GBM | GBM cell and animal models | (Zhao et al., 2022) |
| Upstream regulation | CDK12 axis | CDK12-mediated phosphorylation of MBNL1 | Regulation of vasculogenic mimicry | GBM | GBM cell and animal models | (Liu et al., 2022) |
| Upstream regulation | tRF-1-Ser | Inhibition of MBNL1 nuclear translocation | Associated with oncogenic translation programs | Breast cancer | Breast cancer cell and animal models | (Wan et al., 2024) |
| Upstream regulation | Hypoxia–MBNL1 axis | Hypoxia-induced inhibition of MBNL1 activity | Maintenance of GSC stemness | GBM | GBM stemness models | (Voss et al., 2020) |
| Upstream regulation | CREB1 | CREB1-dependent transcriptional regulation of MBNL1 | Promotion of proliferation and inhibition of apoptosis | GC | Network analysis and cell experiments | (Yu et al., 2022) |
| Upstream regulation | miR-130b-3p | Inhibition of the STAT3/miR-130b-3p/MBNL1 feedback loop | Regulation of tumor angiogenesis | head and neck squamous cell carcinoma (HNSCC) | In vitro and in vivo tumor models | (Li et al., 2022) |
| Downstream regulation | TIAL1/ MYOD1/ Caspase-3 | Suppression of TIAL1–MYOD1–Caspase-9/3 signaling pathway | Reduced metastatic capacity | CRC/ epithelial tumors | Cancer cell functional assays | (Chen et al., 2021) |

**Reference**

Chen, J., Wang, J., Qian, J., Bao, M., Zhang, X., and Huang, Z. (2021). MBNL1 Suppressed Cancer Metastatic of Skin Squamous Cell Carcinoma Via by TIAL1/MYOD1/Caspase-9/3 Signaling Pathways. *Technol Cancer Res Treat* 20**,** 1533033820960755. doi: 10.1177/1533033820960755.

Chen, Y.S., Liu, C.W., Lin, Y.C., Tsai, C.Y., Yang, C.H., and Lin, J.C. (2020). The SRSF3-MBNL1-Acin1 circuit constitutes an emerging axis to lessen DNA fragmentation in colorectal cancer via an alternative splicing mechanism. *Neoplasia* 22(12)**,** 702-713. doi: 10.1016/j.neo.2020.10.002.

Fish, L., Pencheva, N., Goodarzi, H., Tran, H., Yoshida, M., and Tavazoie, S.F. (2016). Muscleblind-like 1 suppresses breast cancer metastatic colonization and stabilizes metastasis suppressor transcripts. *Genes Dev* 30(4)**,** 386-398. doi: 10.1101/gad.270645.115.

Itskovich, S.S., Gurunathan, A., Clark, J., Burwinkel, M., Wunderlich, M., Berger, M.R., et al. (2020). MBNL1 regulates essential alternative RNA splicing patterns in MLL-rearranged leukemia. *Nat Commun* 11(1)**,** 2369. doi: 10.1038/s41467-020-15733-8.

Li, H., Liu, P., Li, D., Wang, Z., Ding, Z., Zhou, M., et al. (2022). STAT3/miR-130b-3p/MBNL1 feedback loop regulated by mTORC1 signaling promotes angiogenesis and tumor growth. *J Exp Clin Cancer Res* 41(1)**,** 297. doi: 10.1186/s13046-022-02513-z.

Liang, C., Chang, Z., Luo, Y., Xu, Y., Chen, A., and Zhang, T. (2022). MBNL1 and MRTF-A form a positive feedback loop in regulating the migration of esophageal cancer cells. *J Cancer Res Ther* 18(5)**,** 1312-1319. doi: 10.4103/jcrt.jcrt_1094_21.

Liu, M., Ruan, X., Liu, X., Dong, W., Wang, D., Yang, C., et al. (2022). The mechanism of BUD13 m6A methylation mediated MBNL1-phosphorylation by CDK12 regulating the vasculogenic mimicry in glioblastoma cells. *Cell Death Dis* 13(12)**,** 1017. doi: 10.1038/s41419-022-05426-z.

Navvabi, N., Kolikova, P., Hosek, P., Zitricky, F., Navvabi, A., Vycital, O., et al. (2021). Altered Expression of MBNL Family of Alternative Splicing Factors in Colorectal Cancer. *Cancer Genomics Proteomics* 18(3)**,** 295-306. doi: 10.21873/cgp.20260.

Ray, D., Yun, Y.C., Idris, M., Cheng, S., Boot, A., Iain, T.B.H., et al. (2020). A tumor-associated splice-isoform of MAP2K7 drives dedifferentiation in MBNL1-low cancers via JNK activation. *Proc Natl Acad Sci U S A* 117(28)**,** 16391-16400. doi: 10.1073/pnas.2002499117.

Seachrist, D.D., Hannigan, M.M., Ingles, N.N., Webb, B.M., Weber-Bonk, K.L., Yu, P., et al. (2020). The transcriptional repressor BCL11A promotes breast cancer metastasis. *J Biol Chem* 295(33)**,** 11707-11719. doi: 10.1074/jbc.RA120.014018.

Tabaglio, T., Low, D.H., Teo, W.K.L., Goy, P.A., Cywoniuk, P., Wollmann, H., et al. (2018). MBNL1 alternative splicing isoforms play opposing roles in cancer. *Life Sci Alliance* 1(5)**,** e201800157. doi: 10.26508/lsa.201800157.

Tang, L., Zhao, P., and Kong, D. (2019). Muscleblind‑like 1 destabilizes Snail mRNA and suppresses the metastasis of colorectal cancer cells via the Snail/E‑cadherin axis. *Int J Oncol* 54(3)**,** 955-965. doi: 10.3892/ijo.2019.4691.

Voss, D.M., Sloan, A., Spina, R., Ames, H.M., and Bar, E.E. (2020). The Alternative Splicing Factor, MBNL1, Inhibits Glioblastoma Tumor Initiation and Progression by Reducing Hypoxia-Induced Stemness. *Cancer Res* 80(21)**,** 4681-4692. doi: 10.1158/0008-5472.Can-20-1233.

Wan, X., Shi, W., Ma, L., Wang, L., Zheng, R., He, J., et al. (2024). A 3'-pre-tRNA-derived small RNA tRF-1-Ser regulated by 25(OH)D promotes proliferation and stemness by inhibiting the function of MBNL1 in breast cancer. *Clin Transl Med* 14(5)**,** e1681. doi: 10.1002/ctm2.1681.

Wang, T., Liu, Q., and Duan, L. (2020). MBNL1 regulates resistance of HeLa cells to cisplatin via Nrf2. *Biochem Biophys Res Commun* 522(3)**,** 763-769. doi: 10.1016/j.bbrc.2019.11.162.

Yan, Y., Kong, X., Jin, X., Bu, J., Ni, B., Rao, Z., et al. (2025). RNA‑binding protein MBNL1 regulates tumor growth, chemosensitivity and antitumor immunity in lung adenocarcinoma by controlling the expression of tumor suppressor RNF125. *Oncol Rep* 54(1). doi: 10.3892/or.2025.8907.

Yu, B., Dai, W., Pang, L., Sang, Q., Li, F., Yu, J., et al. (2022). The dynamic alteration of transcriptional regulation by crucial TFs during tumorigenesis of gastric cancer. *Mol Med* 28(1)**,** 41. doi: 10.1186/s10020-022-00468-7.

Zhao, Y., Song, J., Dong, W., Liu, X., Yang, C., Wang, D., et al. (2022). The MBNL1/circNTRK2/PAX5 pathway regulates aerobic glycolysis in glioblastoma cells by encoding a novel protein NTRK2-243aa. *Cell Death Dis* 13(9)**,** 767. doi: 10.1038/s41419-022-05219-4.
